# Successful Use of Advertisement Pictures to Assist Recall in a Food-Borne Hepatitis A Outbreak in The Netherlands, 2017

**DOI:** 10.1007/s12560-018-9347-3

**Published:** 2018-05-04

**Authors:** Madelief Mollers, Ingeborg L. A. Boxman, Harry Vennema, Ife A. Slegers-Fitz-James, Diederik Brandwagt, Ingrid H. Friesema, Jenny S. Batstra, Margreet J. M. te Wierik

**Affiliations:** 1 National Coordination Centre for Communicable Disease Control, Centre for Infectious Disease Control, National Institute for Public Health and the Environment, Antonie van Leeuwenhoeklaan 9, 3720 BA Bilthoven, The Netherlands; 2 European Programme for Intervention Epidemiology Training (EPIET), ECDC, Tomtebodavägen 11a, 171 65 Solna, Sweden; 3 Laboratory for Feed and Food Safety, Netherlands Food and Consumer Product Safety Authority (NVWA), Catharijnesingel 59, 3511 GG Utrecht, The Netherlands; 4 Infectious Diseases, Research, Diagnostics and Screening, Centre for Infectious Disease Control, National Institute for Public Health and the Environment, Antonie van Leeuwenhoeklaan 9, 3720 BA Bilthoven, The Netherlands; 5 Incidence and Crisis Centre, Netherlands Food and Consumer Product Safety Authority (NVWA), Catharijnesingel 59, 3511 GG Utrecht, The Netherlands; 6 Infectious Diseases, Epidemiology and Surveillance, Centre for Infectious Disease Control, National Institute for Public Health and the Environment, Antonie van Leeuwenhoeklaan 9, 3720 BA Bilthoven, The Netherlands

**Keywords:** HAV, Soft fruit, Raspberries, Virus, Outbreak

## Abstract

This study describes an outbreak investigation of 14 hepatitis A cases in the Netherlands. The hepatitis A virus (HAV) genotype IB sequences in cases were highly similar (459/460 nt). The origin of strains could be narrowed to Bulgaria based on information from EPIS-FWD. As an association with consumption of soft fruit was suspected, a case–control study was initiated using a questionnaire and a list of pictures of soft fruit available at the supermarket chain involved. Twelve out of 13 cases consumed a specific frozen raspberry/blueberry product shown on the list (OR 46.0, 95% CI 5.0–27). In multivariable regression analysis this product was the only risk factor (aOR 26.6, 95% CI 2.0–263). Laboratory analyses could not demonstrate HAV-RNA in batches that had been on the market in the incubation period of patients. Trace back of frozen fruit showed that raspberries had been traded by a producer in Bulgaria. After withdrawal of the product from the supermarket no new cases were reported. Use of advertisement pictures of consumed food was helpful in this investigation. Suspicion of the source was strengthened by data from molecular typing and food trace back activities, underlining the importance of good (inter)national cooperation between public health and food safety organisations.

## Background

Hepatitis A virus (HAV) is a faecal-orally transmitted pathogen causing acute self-limiting hepatitis. Risk factors for infection include exposure to infected persons, contaminated surfaces, food or water. Hepatitis A can re-emerge in regions such as North America and Western Europe, where it is not endemic anymore, affecting mostly adults, with more severe course of infection (WHO/FAO 2008).

The notification rate in European Union (EU)/European Economic Area (EEA) member states for Hepatitis A shows a steadily decreasing trend over the past decades (ECDC 2016). As a result introduction of HAV-contaminated food in EU/EEA countries may lead to diffuse outbreaks that are geographically and temporally dispersed. Between 2007 and 2012, EFSA and ECDC reported 14 hepatitis A outbreaks in which there was strong evidence of food being the infection vehicle (EFSA 2014). More recently, three multinational outbreaks of hepatitis A affecting EU/EEA countries were reported with suspected transmission through soft fruit (Gillesberg Lassen et al. 2013; EFSA 2014; Sane et al. 2015). In the Netherlands the incidence rate for hepatitis A is usually low with on average 0.8 cases per 100.000 population per year in 2007–2016 (range 0.5–1.6) (RIVM 2016). The incidence rate increased in 2017 due to an outbreak of hepatitis A among men who have sex with men (MSM) which started in 2016 (Freidl et al. 2017). Being a notifiable disease, cases are reported in a national electronic registration system for infectious diseases (Osiris) including demographic and epidemiological data (Petrignani et al. 2014). Serum or faecal samples from ca. 70% of notified hepatitis A cases are sent to the National Institute for Public Health and Environment (RIVM) for genotyping. Surveillance is intensified for cases with no travel history to endemic countries and an unknown source of infection, by administering a hypothesis generating questionnaire (Petrignani et al. 2014).

The present study describes the outbreak investigation in which molecular genotyping, a case–control study using a list of food pictures, food analysis, and trace back led to recall of the suspected food product.

## Outbreak Detection

In April 2017, three cases of hepatitis A were notified by the same Public Health Service (PHS), who had an onset of illness within 8 days. None of them had a history of travel to endemic countries or MSM contact. However, cases were living in the same neighbourhood and bought food at the same supermarket store. A local outbreak investigation was inconclusive.

**Fig. 1:**
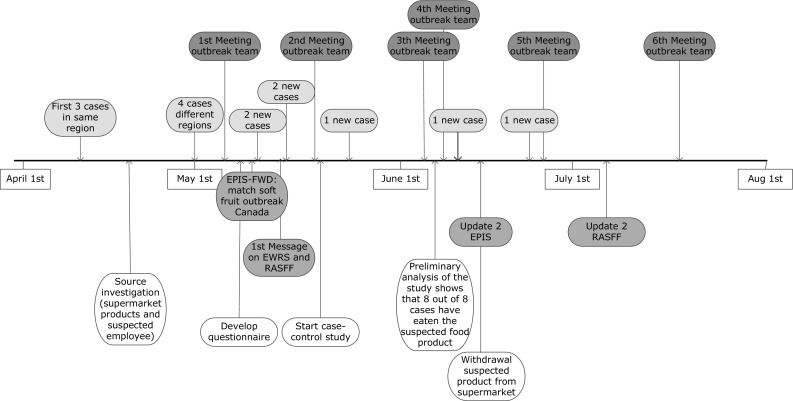
Timeline of the outbreak investigation on a possible food-borne hepatitis A outbreak with 14 cases in the Netherlands, 2017 (cases are shown by reporting date in contrast to Fig. 2 epicurve)

A national outbreak investigation was initiated when a fourth and fifth case were reported from two different geographical regions and the RIVM showed that all cases had been infected by an identical genotype IB strain (see Fig. 1, timeline).

**Fig. 2:**
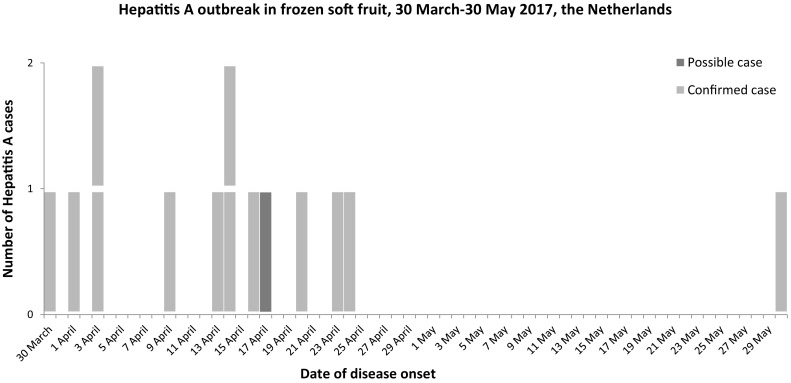
Epicurve of a hepatitis A outbreak related to frozen soft fruit with date of disease onset of cases between 30 March–30 May 2017, the Netherlands

## Methods

### Case Finding

HAV in serum or faecal samples was genotyped as earlier described (Grinde et al. 1997; Stene-Johansen et al. 2007). Cases were identified as a cluster case if they had been infected by a genotype IB strain sharing at least 459/460 nucleotides with the RIVM-HAV17-090 outbreak strain from 30th of March onwards.

### Controls

Controls were recruited from travel clinics of the PHS and matched the demographic region of the cases. The aim was to include four controls for each case. If the travel clinic was visited by a couple, family or group, only one person was included in the study. No further matching between cases and controls was performed. Only controls that had never been HAV-vaccinated, were included.

### Questionnaire and Statistical Analyses

An earlier described hypothesis generating questionnaire (EFSA 2014) was modified and focused on soft fruit consumption (available upon request [in Dutch]). From the supermarket chain which was visited by all cases for their groceries, a list of 24 advertisement pictures of products available on the website of the specific supermarket chain was used. The list of advertisement pictures consisted of fresh raspberries and blackberries, cartons or plastic bags of frozen soft fruit, and bottles of juices, smoothies, dressings, and yoghurt drinks with fruit containing blackberries and/or raspberries. This list was based on results from the national registration questionnaire (Osiris) indicating frequent consumption of blackberries and/or raspberries by cases (10/14). Cases and controls filled in the questionnaire and the image list either by mail or in the presence of an officer of the regional PHS. Data were analysed using a univariable and multivariable logistic regression model (SAS version 9.4 SAS Institute Inc., USA).

### Food Analyses

Twenty-seven cartons containing a mix of frozen blueberries and raspberries were collected by inspectors of the Netherlands Food and Consumer Product Safety Authority (NVWA) and transported and stored frozen until analysis at the Laboratory for Feed, Food and Product Safety of the NVWA in Wageningen, the Netherlands. The 27 cartons represented 13 different batches known to be present on the market in the incubation period of the patients, one to four cartons per batch. Of each carton three subsamples of 25 grammes were analysed. Analysis for the presence of HAV was performed according to ISO15216-2 under accreditation of the Dutch Council for Accreditation. Prior to reverse-transcription real-time polymerase chain reaction (RT qPCR) all RNA samples were treated to reduce inhibitory substances and increase the detectability of the target RNA using the OneStep PCR Inhibitor Removal Kit (Zymo Research) (Boxman et al. 2016a).

## Results

### Descriptive Epidemiology

The outbreak cluster, including one secondary case, consisted of seven males and seven females, with ages ranging from 16 to 60 years. Nine cases had been infected by the HAV IB outbreak strain RIVM-HAV17-090 (460 nt). Four cases had been infected with nearly identical HAV variants with two different single nucleotide differences. Another case was epidemiologically linked to the cluster, being the index case for a confirmed secondary case, but a diagnostic sample was not available for genotyping. The onset of illness ranged between 30 March and 30 May 2017, but for most cases the onset was within the first month of this period (see Fig. 2, Epicurve). No information on the severity of illness was known, but 5 out of 13 cases (38%) were admitted to hospital. None of the patients died.

### Genotyping and International Enquiry

The outbreak genotype IB strain (RIVM-HAV17-090) was typical for samples from the northern part of the Eastern Mediterranean region (RIVM 2017). The sequence was shared on EPIS-FWD (epidemic intelligence information system for food- and waterborne diseases) (5 May 2017) to be able to identify more cases and clues. This revealed a match with an identical sequence detected in cases of a HAV outbreak associated with consumption of mixed frozen berries in Canada, May–June 2016 (PHAC 2016). The Canadian outbreak was shown to be associated with HAV-contaminated blackberries from Bulgaria by viral analyses. In addition, the origin of the HAV strain was confirmed independently with information from Italy through EPIS-FWD. It was shown that the strain had been detected earlier in clinical samples of patients from Bulgaria. The international community within the EU was notified through EWRS (early warning and response system) (10 May 2017) and RASFF (food and feed safety alerts) (11 May 2017).

### Risk Factors Analyses

Thirteen of the 14 cases, including the non-confirmed case, and 29 controls completed the questionnaire and image list. The median age of the cases was 29 years (range 16–60 years) compared to 27 years (range 20–54 years) of the controls. Fifty-four percent were men compared to 45% of controls.

Based on the questionnaires, frozen mixed fruit was consumed by all cases, but by only 24% of the controls (OR 38.5, 95% CI 5.0–853). In addition, there was a clear association with consumption of frozen raspberries, mixed frozen fruit or consumption of smoothies made of mixed fruit (Table 1), but for none of the 94 other food items.

**Table 1:** Food items consumed within 6 weeks before onset of illness by hepatitis A cluster cases (*n* = 13) in comparison to controls (*n* = 29), the Netherlands, April–May 2017

	Cases (*n* = 13)	Controls (*n* = 29)	OR	95% CI	aOR^a^	95% CI
Questionnaire						
Frozen mixed fruit^b^	13 (100%)	7 (24%)	38.5^c^	(5.0–853)	^d^	
Frozen raspberries	9 (69%)	8 (26%)	5.6	(1.3–23.6)	NS	
Mixed fruit smoothie^b^	4 (31%)	1 (3%)	12.4	(1.2–126)	NS	
Image list^e^						
Carton of frozen blueberries/raspberries	12 (92%)	6 (21%)	46.0	(5.0–427)	26.6	(2.0–263)
Carton of frozen fruits^f^	9 (69%)	3 (10%)	19.5	(3.6–104)	NS	
Bag of frozen fruits^f^	5 (38%)	2 (7%)	8.4	(1.4–52.1)	NS	

*NS* not significant

^a^aOR = adjusted OR obtained from the multivariable model

^b^Any fruit

^c^The OR was calculated by hand by adding 1 extra person to each cell in a 2 × 2 table, because all cases had consumed this product

^d^Not possible to calculate

^e^Results for one case are missing

^f^Raspberries, blueberries, blackberries, strawberries, cherries and red currants

Twelve out of 13 cases recalled to have consumed one particular raspberry/blueberry product shown on the advertisement picture list (OR 46.0, 95% CI 5.0–427). Univariable logistic regression analyses further identified a blend of frozen fruit as risk factor, which was a blend of raspberries, blackberries, strawberries, cherries, blueberries and red currants (Table 1). In multivariable regression analysis, the specific carton with mixed frozen blueberries and raspberries was the only remaining risk factor (aOR 26.6, 95% CI 2.0–263).

### Trace Back and Viral Analyses of Soft Fruit Samples

The suspected raspberry/blueberry product was exclusively sold at shops of one supermarket chain in the Netherlands. Requested trace back data for this particular product in the incubation period of the cases (half February until half April) revealed that 24 batches had been on the market, and that in this particular period raspberries were traded by a producer in Bulgaria whereas the blueberries were traded by producers in other countries. Herewith the raspberries became the suspected food type, as the country trading the raspberries corresponded to the most likely country origin of the outbreak HAV strain. No common suppliers could be identified between this and the Canadian outbreak (PHAC 2016), personal communication).

Analyses of samples were restricted to those batches that had been on the market in the exposure period of the cases. As raspberries were the suspected fruit type in the mix, three subsamples of raspberries only were weighed from each of the 27 cartons and analysed. None of the 81 subsamples tested positive for HAV-RNA. Quality assurance parameters showed an extraction efficiency of the added murine norovirus process control of 8.2 ± 4.7% (range 4–16%) and no inhibition in the RNA samples was seen.

Based on the epidemiological findings, specified batches to which cases most likely had been exposed were classified as potentially unsafe for human consumption by the NVWA. The retailer subsequently initiated a total withdrawal of the product (12 June 2017) for replacement with batches coded with later production dates. No new cluster cases were identified thereafter. International alerts and sharing of sequences in the international databases did not result in identification of additional cases.

## Discussion

Combined analyses of data has led to the conclusion that frozen raspberries from Bulgaria were the most likely source for this food-borne hepatitis A virus outbreak. Molecular typing showed that cases clustered by being infected by (nearly) the same (459/460 nt) HAV strain. This strain was most likely associated with Eastern Mediterranean countries, but the geographical association could be further narrowed to Bulgaria by typing information of cases from Bulgaria (EPIS-FWD) as well as cases in a Canadian outbreak associated with Bulgarian fruit (PHAC 2016). Our case–control study identified a raspberry/blueberry product, containing Bulgarian raspberries, consumed by 92% of the cases (aOR 26.6, 2–263).

Identification of cluster cases that are geographically and temporarily dispersed strongly depend on national surveillance, which in the Netherlands has a high coverage for typing of diagnostic samples (70%) (Petrignani et al. 2014). Through this surveillance system, several, mainly small, food-borne hepatitis A outbreaks have been identified in recent years, e.g. outbreaks associated with semi-dried tomatoes (Petrignani et al. 2010; Fournet et al. 2012), soft fruit (EFSA 2014), as well as mussels (Boxman et al. 2016b). In the present outbreak, cases were identified as a cluster despite the large (inter)national outbreak in MSM that occurred at the same time (Freidl et al. 2017), as outbreak strains were different.

The fact that all patients went to the same supermarket chain for their groceries, led to the idea of adding a list of the supermarket’s advertisement pictures of raspberry and blackberry containing products. The use of pictures has earlier shown to be helpful in recall of consumption of suspected products (Guzman-Herrador et al. 2014). Also in the present study, the picture list had a clear added value, as it identified one specific product, i.e. the brand and the composition, whereas the questionnaire only identified mixed frozen fruit as a risk factor. Especially the fact that almost all cases ate the same product was strong. Trace back data indicated the raspberries in the box of frozen raspberries and the box of mixed frozen fruit originated from the same producer in Bulgaria.

Interestingly, most cases recalled consumption of the frozen soft fruit products five or more times a week. It suggests that such consumption patterns increased the risk of infection in these cases, particularly when the contamination of these raspberries is low, possibly too low to be detected by viral analyses and also maybe too low to infect a person after a single exposure only. Due to low numbers we were not able to explore this possible dose–response association any further.

Detection of the viral strain in the implicated food is difficult and often hampered by absence of leftovers, absence of an adequate method for extraction or heterogeneous distribution of low amounts of virus in different batches. In this outbreak the contaminated batch(es) might even not have been among the tested batches. Only samples of 13 of the 24 batches that were present on the market during the incubation period of the cases were available for testing, also indicating the high through-put of these products on the market.

It is expected that outbreaks related to soft fruit keep occurring and being reported (Tavoschi et al. 2015). The berry production has experienced an increase in the last decade and this food commodity often receives no or only minimal (industrial) processing. The FAO/WHO (FAO/WHO 2012) has produced a Codex guideline for better hygienic practices to reduce the illness by viral contaminated food, including specific recommendation for berries production in a separate Annex. Food business operators and trade associations have an important role in providing specific instructions and training of personnel for control of viruses. In countries where HAV is endemic, risk communication should focus on preventive measures of which hand washing after using the toilet at the farm level and providing good equipped sanitary facilities to the amount of seasonal influx of workers seems the most effective (FAO/WHO 2008). Other options, already implemented by some countries is to harvest berries mechanically, or to boil frozen berries before use (Gillesberg Lassen et al. 2013), but boiling does alter the taste and texture.

In conclusion, the retailer was willing to recall the implicated product by the strong epidemiologically link of the cases with frequent consumption of one specific product. Since the recall, no new cases with the same genotype were reported in the Netherlands. The present outbreak investigation showed the strength of advertisement pictures in addition to questionnaires to assist recall of implicated food in outbreak studies. Data from molecular typing and food trace back activities strengthened the link to the suspected source. This underlines the importance of good (inter)national cooperation between public health and food safety organisations to fully profit from each other’s expertise.
